# Genome-Wide Association Studies and fine-mapping of genomic loci for n-3 and n-6 Polyunsaturated Fatty Acids in Hispanic American and African American Cohorts

**DOI:** 10.21203/rs.3.rs-2073736/v1

**Published:** 2023-02-24

**Authors:** Chaojie Yang, Jenna Veenstra, Traci Bartz, Matthew Pahl, Brian Hallmark, Yii-Der Ida Chen, Jason Westra, Lyn Steffen, Christopher Brown, David Siscovick, Michael Tsai, Alexis Wood, Stephen Rich, Caren Smith, Timothy O'Connor, Dariush Mozaffarian, Struan Grant, Floyd Chilton, Nathan Tintle, Rozenn Lemaitre, Ani Manichaikul

**Affiliations:** University of Virginia; Dordt University; University of Washington; Children's Hospital of Philadelphia; University of Arizona; Lundquist Institute for Biomedical Innovation at Harbor-UCLA Medical Center; Dordt University; University of Mineesota; Perelman School of Medicine, University of Pennsylvania; 42. The New York Academy of Medicine, New York, NY; University of Minneosta; Baylor College of Medicine; University of Virginia; Tufts University; University of Maryland, Baltimore; Tufts University; Children's Hospital of Philadelphia Research Institute; University of Arizona; Fatty Acid Research Institute; Cardiovascular Health Research Unit, University of Washington; University of Virginia School of Medicine

## Abstract

Omega-3 (n-3) and omega-6 (n-6) polyunsaturated fatty acids (PUFAs) play critical roles in human health. Prior genome-wide association studies (GWAS) of n-3 and n-6 PUFAs in European Americans from the CHARGE Consortium have documented strong genetic signals in/near the *FADS* locus on chromosome 11. We performed a GWAS of four n-3 and four n-6 PUFAs in Hispanic American (n = 1454) and African American (n = 2278) participants from three CHARGE cohorts. Applying a genome-wide significance threshold of *P* < 5 x 10^−8^, we confirmed association of the *FADS* signal and found evidence of two additional signals (in *DAGLA* and *BEST1*) within 200 kb of the originally reported *FADS* signal. Outside of the *FADS* region, we identified novel signals for arachidonic acid (AA) in Hispanic Americans located in/near genes including *TMX2, SLC29A2, ANKRD13D* and *POLD4*, and spanning a >9 Mb region on chromosome 11 (57.5Mb ~ 67.1 Mb). Among these novel signals, we found associations unique to Hispanic Americans, including rs28364240, a *POLD4* missense variant for AA that is common in CHARGE Hispanic Americans but absent in other race/ancestry groups. Our study sheds light on the genetics of PUFAs and the value of investigating complex trait genetics across diverse ancestry populations.

## Introduction

Omega-3 (n-3) and omega-6 (n-6) polyunsaturated fatty acids (PUFAs) are critical structural components of cell membranes, which can influence cellular activities by promoting the fluidity, flexibility, and the permeability of a membrane.^1-3^ Additionally, PUFAs affect a variety of other biological processes and molecular pathways, including modulating membrane channels and proteins, regulating gene expression through nuclear receptors and transcription factors, and conversion of the PUFAs themselves into bioactive metabolites.^4^ Levels of circulating PUFAs and long chain (≥ 20 carbons) PUFAs (LC-PUFAs) are associated with reduced risk of cardiovascular disease^5,6^, type 2 diabetes mellitus^7^, cognitive decline^8^, Alzheimer's disease^9^, metabolic syndrome^10^ and breast cancer^11^, as well as all-cause mortality.^12^

**Figure 1:**
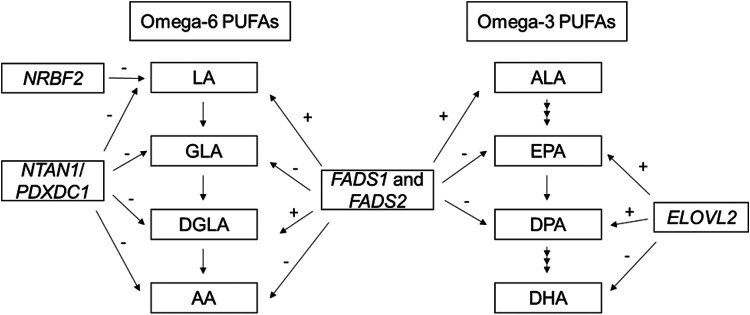
PUFAs metabolic pathway and summary of genome-wide association from previous CHARGE GWAS of n-3 and n-6 PUFAs in European Americans Figure 1 shows the summary of genome-wide association from previous CHARGE GWAS of n-3 and n-6 PUFAs in European Americans. + and − signs indicate the direction of the associations for the minor allele of most significant SNP at each locus. The SNPs used to determine the directions of effect at each locus: *FADS1* and *FADS2*: rs174547 (ALA, DPA, LA, GLA, DGLA and AA); rs174538 (EPA) *ELOVL2*: rs780094 (DPA); rs3798713 (EPA); rs2236212 (DHA) *NTAN1/PDXDC1*: rs16966952 (LA, GLA, DGLA and AA) *NRBF2*: rs10740118 (LA).

PUFAs and LC-PUFAs are characterized by the position of the first double bond from the methyl terminal (omega; ω; or n – FAs) and fall into two primary families, n-3 and n-6. The most abundant n-3 PUFAs are alpha-linolenic acid (ALA), eicosapentaenoic acid (EPA), docosapentaenoic acid (DPA) and docosahexaenoic acid (DHA), while the primary n-6 PUFAs are linoleic acid (LA), gamma-linolenic acid (GLA), dihomo-γ-linolenic acid (DGLA) and arachidonic acid (AA). ALA and LA are essential n-3 and n-6 PUFAs consumed from the diet and these then can be converted to more unsaturated LC-PUFAs through a set of desaturation and elongation enzymatic steps. For example, DGLA and AA can be synthesized from LA, while EPA, DPA and DHA can be produced from ALA (Fig. 1). The precursors LA and ALA are essential fatty acids that must be provided by the diet. Due to the lower abundance of ALA in Western diets and the inefficiency of conversion of ALA to longer chain n-3 LC-PUFAs such as EPA and DHA, dietary intake of these via fatty fish or marine oil supplementation is often recommended.^13,14^

Previous studies have shown that African ancestry populations have higher circulating levels of LC-PUFAs compared to European Americans.^15^ These large differences can be explained in part by variation in the allele frequencies of *FADS* variants associated with different biosynthetic efficiencies in these two populations.^16^ Mathias *et al.* also revealed that African Americans have significantly higher levels of AA and lower levels of the AA precursor DGLA, and that *FADS1* variants were significantly associated with AA, DGLA and the AA/DGLA ratio in a sample of fewer than 200 African Americans from the GeneSTAR study.^15^ In addition, African ancestry populations have higher frequencies of the “derived” *FADS* haplogroup (represented by the variant rs174537 allele G)^17^ that is associated with more efficient conversion for PUFAs.^16^ In contrast, Amerind ancestry Hispanic populations have higher frequencies of the “ancestral” *FADS* haplogroup (represented by rs174537 allele T) that has a reduced capacity to synthesize PUFAs. Accordingly, we demonstrated that higher global proportions of Amerind ancestry are associated with lower levels of PUFAs in Hispanic populations.^17^

Genome-wide association studies (GWAS) of n-3 and n-6 PUFAs were performed by the CHARGE consortium in European ancestry (EUR) participants.^18-20^ The CHARGE GWAS of n-3 PUFAs in 8,866 European Americans identified genetic variants in/near *FADS1* and *FADS2* associated with higher levels of ALA and lower levels of EPA and DPA, as well as SNPs in *ELOVL2* associated with higher EPA and DPA and lower DHA. The CHARGE GWAS of n-6 PUFAs in 8,631 European Americans confirmed that variants in the *FADS* gene cluster were associated with LA and AA, and it revealed that variants near *NRBF2* were associated with LA and those in *NTAN1* were associated with LA, GLA, DGLA, and AA (Fig. 1). In the Framingham Heart Offspring Study, variants in/near *PCOLCE2, LPCAT3, DHRS4L2, CALN1 FADS1/2*, and *ELOVL2* were associated with PUFAs in European ancestry participants.^21,22^ Collectively, these studies played an important role in identifying the genetic associations of n-3 and n-6 PUFAs in European ancestry populations.

To address the paucity of GWAS of PUFAs in non-European ancestry cohorts, we performed a metaanalysis of genome-wide association studies for n-3 and n-6 PUFAs for Hispanic American (HIS) and African American (AFA) participants from three CHARGE consortium cohorts: the Multi-Ethnic Study of Atherosclerosis (MESA), the Cardiovascular Health Study (CHS) and the Framingham Heart Study (FHS) Omni cohort. The major goals of the study were (1) to examine whether the major loci identified in European Americans are shared across race/ancestry groups, and (2) to examine evidence for genetic association unique to HIS and AFA populations. As GWAS approaches are not sufficient to identify the causal variants and determine the number of independent signals, especially in the context of long stretches of linkage disequilibrium (LD) within the *FADS* locus^15,23^, we conducted statistical fine-mapping^24^ to identify the most likely causal variants within each n-3 and n-6 PUFA-associated locus. We performed cross-ancestry replication analysis in CHARGE and MESA, with validation using the multiancestry GWAS of lipids from the Global Lipids Genetics Consortium (GLGC).^25^ Subsequently, we performed integrative analysis leveraging gene expression data from MESA^26,27^ and the Genotype-Tissue Expression (GTEx) project^28^ to identify genes that could contribute to our identified genetic association results. Finally, we examined open chromatin defined by ATAC-seq to determine the impact and physical contact of the identified variants with nearby genes. Our study demonstrates the vital importance of diverse ancestry genetic studies for the study of complex traits, and particularly for metabolites that have been subject to evolutionary pressures and are closely regulated by specific protein-coding genes.

## Results

### Participant characteristics

The participants in the meta-analysis of GWAS for PUFAs included 1,454 HIS and 2,278 AFA unrelated participants (Table 1; fatty acid levels are expressed as the percentage of total fatty acids throughout the entire manuscript). There were some differences in the distributions of fatty acid levels observed across cohorts, which were likely due to the sources of biospecimens for the assays (plasma phospholipids for MESA and CHS versus erythrocytes for FHS). For example, mean levels of DPA varied from 0.85% (CHS: plasma phospholipids) to 2.54% of total fatty acids (FHS: erythrocytes) in AFA and AA from 11.01% (MESA: plasma phospholipids) to 16.56% (FHS: erythrocytes) in HIS (Table 1). In addition, n-6 PUFAs, especially LA and AA, have relatively higher mean levels than n-3 PUFAs in all cohorts (Table 1).

Table 1 shows the participant characteristics of the Hispanic Americans and African Americans from each cohort (MESA, CHS and FHS). Data are presented as n (%) for binary measures or median [IQR] for continuous measures. Summary statistics are reported for the subset of individuals with data available for at least one of the fatty acid traits examined in genetic analyses. Fatty acids were measured in plasma phospholipids in MESA and CHS and in erythrocytes in FHS.

Regardless of whether the fatty acids were measured in plasma phospholipids or erythrocytes, AFA populations had higher levels of AA and elevated ratios of AA to DGLA and AA to LA relative to Hispanic populations. This result would be expected given the frequency differences in the derived (efficient) to ancestral (inefficient) *FADS* haplogroups between these two populations. As expected, due to the lower levels of dietary ALA relative to LA entering the biosynthetic pathway, levels of n-3 LC-PUFAs including EPA, DPA and DHA were significantly lower than the n-6 LC-PUFA, AA. Additionally, African Americans had higher levels of n-3 LC-PUFAs than Hispanic Americans, again likely due to differences in the ratio of the derived to ancestral *FADS* haplogroups. These differences are similar to those observed examining the same PUFAs and LC-PUFAs and ratios when comparing African Americans and European Americans.^15,29^

### Confirmation Of Top Variants Identified In Prior Charge Eur Gwas Of Pufas

We began by examining associations of seven known PUFA-associated signals from CHARGE EUR in our current study of CHARGE HIS and AFA. Multiple variants identified by previous CHARGE EUR GWAS metaanalyses were also identified in CHARGE HIS (*FADS1/2* region: rs174547 and rs174538, *PDXDC1* variant: rs16966952 and *GCKR* variant: rs780094) and AFA (*FADS1/2* region: rs174547, *PDXDC1* variant: rs16966952, *GCKR* variant: rs780094 and *ELOVL2* variant: rs3734398) after adjusting for multiple testing for the number of variants examined across the eight PUFAs (P < 0.05/8 = 0.006) (**Table S1**). The directions of effect observed in HIS and AFA for these variants were consistent with those reported for European ancestry populations in prior CHARGE GWAS meta-analyses of n-3 and n-6 PUFAs (**Table S1**).

### Gwas And Fine-mapping Identify Novel Pufa-associated Genetic Signals In Charge His And Afa

Based on a genome-wide significance threshold of *P* < 5 x 10^−8^, our complete GWAS of n-3 and n-6 PUFAs identified associations on chromosomes 11, 15 and 16 in CHARGE HIS (Table 2) and chromosomes 6, 7, 10 and 11 in CHARGE AFA (Table 3). For regions with more than one genome-wide significant variant, we applied statistical fine-mapping to identify the independent putative causal signals (credible sets) for each genome-wide significant locus. We carried out these analyses separately for our CHARGE HIS and CHARGE AFA GWAS meta-analysis results.

Table 2 shows the signals (credible sets) of putative causal variants identified at each chromosome for each PUFAs from SuSiE in the HIS. All variant positions are presented based on Human Genome Build 37. Variants previously documented in the CHARGE GWAS meta-analysis of n-3 and n-6 PUFAs were considered known prior to the current meta-analysis. Additionally, those variants demonstrating linkage disequilibrium (LD) R-squared > 0.2 with one or more previously reported GWAS variants were considered known. The remaining variants that were not in LD with known GWAS variants were considered novel in the current study. There was only one genome-wide significant variant on chromosome 15 for DGLA (rs57112407) in HIS, this signal was not carried forward for fine-mapping.

Table 3 shows the signals (credible sets) of putative causal variants identified at each chromosome for each PUFAs from SuSiE in AFA. All variant positions are presented based on Human Genome Build 37. Variants previously documented in the CHARGE GWAS meta-analysis of n-3 and n-6 PUFAs were considered known at the current meta-analysis. Additionally, those variants demonstrating linkage disequilibrium (LD) R-squared > 0.2 with one or more previously reported GWAS variants were considered known. The remaining variants that were not in LD with known GWAS variants were considered novel in the current study. There was only one genome-wide significant variant on chromosome 10 for DHA (rs114622288) in AFA, this signal was not carried forward for fine-mapping.

We identified multiple independent putative causal signals for the PUFA traits [AA: 8 signals (credible sets); ALA: 1; DGLA: 5, DPA: 2; EPA: 1; GLA: 1; LA: 6] in HIS and [AA: 5; DGLA: 2, DPA: 2, LA: 1] in AFA (Table 2, Table 3, **Table S2 and Table S3**). We examined the overlap of signals identified from fine-mapping in HIS versus AFA. We observed that the credible sets were generally smaller in AFA (average number of variants in credible set: HIS:3.4; AFR:2.2) possibly driven by the lower average LD in AFA.

Among the independent credible sets identified, most were novel associated signals within a +/− 5 Mb region of the previously reported FADS signal on chromosome 11 (Tables 2-3). Examining all the signals for PUFAs in HIS and AFA, we observed that the lead signal (reflecting the strongest evidence of association) on chromosome 11 represents the *FADS* signal reported in the previous GWAS.^20^ For example, rs174547, the *FADS1* variant reported in the previous CHARGE EUR GWAS, is one of the variants in the first credible set for AA in HIS.^19,20^ In addition to the known *FADS* signals, we also observed multiple novel independent signals at other regions of chromosome 11 for PUFAs [AA: 5 novel signals (credible sets) and LA: 3] in HIS, for example, in/near *ANKRD13D, TMX2, POLD4* and *SLC29A2* and spanning a long range (57.5Mb ~ 67.1 Mb) on chromosome 11 for AA in HIS (Table 2). Additionally, we observed several novel independent signals on other chromosomes showing associations with the PUFA traits in AFA [AA: 1 novel signal on chromosome 7 and DPA: 1 on chromosome 6] (Table 3).

#### Additional independent PUFA-associated signals on chromosome 11 demonstrate chromatin contacts with FADS and other genes

**Figure 2:**
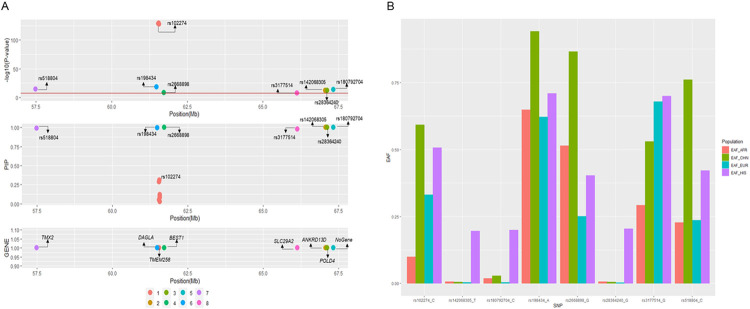
Summary of signals (credible sets) identified in association with AA on chromosome 11 in Hispanic Americans. Figure 2 shows the information (Panel A: GWAS_Pvalue, Posterior Inclusion Probability, Nearest genes and Panel B: Allele effect frequencies) of the putative causal variants of each signal (credible set) showing the association with AA on chromosome 11 in Hispanic Americans. In panel A, the upper panel shows the *P*-value of the putative causal variants of each signal (credible set) on chromosome 11 from GWAS; middle panel shows the Posterior Inclusion Probability (PIP) of the putative causal variants from statistical fine-mapping using SuSIE; bottom panel shows the Gene near/in the putative causal variants of each signal. Panel B shows the effect allele frequencies across four race/ethnic groups (African American, European, Hispanics and Chinese) of the most significant putative causal variant from each signal (credible set).

**Figure 3:**
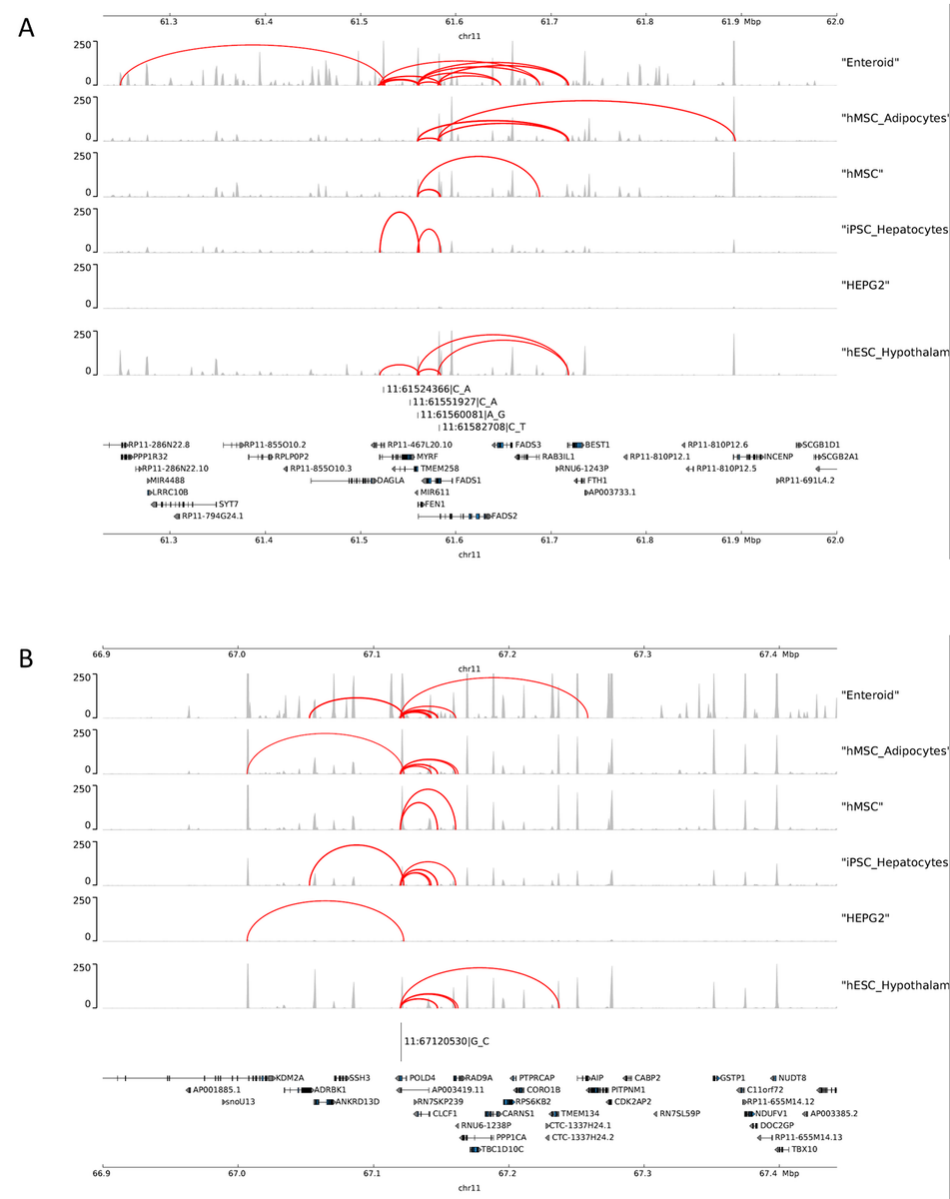
Chromatin contact analysis of selected genome-wide significant variants identified on Chromosome 11 Figure 3 shows the chromatin contact between the causal variants within the signals (Panel A: *FADS* region and Panel B: *POLD4*region) located in open chromatin defined by ATAC-seq with gene promoters defined by Promoter Capture C in multiple metabolic-relevant cell types, including: human mesenchymal stem cells (hMSC), which were also differentiated in vitro to adipocytes (hMSC_Adipocytes), induced pluripotent stem cell derived Hepatocytes (iPSC_Hepatocytes), embryonic stem cell derived Hypothalamic Neurons (hESC_HypothalamicNeurons), Enteroids, and HepG2s.

While prior studies have represented the *FADS* signal as primarily just one signal,^19,20^ our study demonstrates numerous independent signals within the *FADS* region (+/−1 Mb of the top variant, rs107724) (Fig. 2A). We examined this region to identify the subset of variants that may affect cis-regulatory elements in physical contact with nearby genes. Four variants within the credible sets in this region were located in regions of open chromatin defined by ATAC-seq and were in contact with gene promoters defined by Promoter Capture C in multiple metabolic-relevant cell types (human mesenchymal stem cells [hMSC], adipocytes derived from in vitro from the hMSC [hMSC_Adipocytes], induced pluripotent stem cell derived Hepatocytes [iPSC_Hepatocytes], embryonic stem cell derived Hypothalamic Neurons [hESC_HypothalamicNeurons], Enteroids, and HepG2s). Almost all of the interactions we detected were bait-to-bait interactions, meaning that they reflected physical contact between promoters of two different genes (**Table S4**). For example, the region surrounding rs2668898 near *BEST1* showed evidence of physical contact with the *TMEM258, FADS1* and *FADS2* region in multiple cell types and *TMEM258* region also showed evidence of physical contact with the *FADS1* and *FADS2* region (Fig. 3A **and Table S4**). Besides the *FADS* region, we further found evidence of physical contact between *POLD4* and *ANKRD13D*(Fig. 3B **and Table S4**), corresponding to the regions surrounding two signals identified in fine-mapping of AA in HIS (Fig. 2A).

#### Three novel signals on chromosome 11 identified in HIS show evidence of cross-ancestry replication or validation

We examined evidence of cross-ancestry replication for signals identified in our present GWAS of CHARGE HIS and AFA by examining evidence of genetic association in European Americans (CHARGE EUR and MESA EUR), African Americans (CHARGE AFA), Hispanic Americans (CHARGE HIS) and Chinese Americans (MESA CHN). Replication analysis was performed with multiple testing correction (HIS: *P* < 0.05/19 signals = 0.0026 and AFA: *P* < 0.05/11 signals = 0.004).

As noted previously, the first credible set identified in our present GWAS of HIS and AFA for each trait (reflecting the strongest evidence of association) generally coincided with the region of chromosome 11 reported in prior CHARGE GWAS efforts. These signals showed evidence of genetic association in European Americans, as well as across race/ancestry groups. For example, rs102274 for AA was replicated in the MESA EUR, CHARGE AFA and MESA CHN groups (MESA EUR: *P* = 1.04 x 10^−151^, CHARGE AFA: *P* = 2.36 x 10^−47^, MESA CHN: *P* = 8.75 x 10^−92^) (**Table S5**).

Additionally, one novel signal was also replicated across race/ancestry groups (Table 4). *LRP4* variant rs11039018 in credible set 5 for LA was replicated in the CHARGE AFA (CHARGE AFA: *P* = 1.90 x 10^−13^).

Table 4 shows the novel putative causal variants in each signal (credible set) identified from Fine-mapping for PUFAs with replication and validation evidence in HIS. Variants that weren’t previously documented in the CHARGE GWAS meta-analysis of n-3 and n-6 PUFAs and weren’t in LD with known GWAS variants were considered novel in the current study.

Some of the novel signals without cross-ancestry replication demonstrated large differences in allele frequencies across groups. For example, the effect allele frequency of rs28364240, a *POLD4* missense variant in credible set 3 for AA in Hispanics, is 0.204 in our CHARGE HIS group, but close to zero in the other race/ancestry groups examined (EUR: 0.003, AFR: 0.007, CHN: 0.005) (Fig. 2B **and Table S5**) and the effect allele frequency of rs142068305, a *ANKRD13D* intron variant, is 0.196 in our CHARGE HIS group while 0.007, 0.004 and 0.005 in AFR, EUR and CHN, respectively. These results suggest evidence of genetic association signals unique to HIS or other groups carrying Amerindian ancestry or admixture.

As some variants could not be interrogated using independent GWAS of PUFA traits, given those studies’ focus on specific race/ancestry groups which may not include our variants of interest and/or limited sample sizes, we performed validation analyses using the results of multi-ancestry GWAS of lipid levels from the GLGC including ~ 1.65 million individuals from five genetic ancestry groups (admixed African or African, East Asian, European, Hispanic and South Asian). We focused on the most significant putative causal variants from each credible set and applied multiple testing correction for the number of validated variants (HIS: *P* < 0.05/19 = 0.0026 and AFA: *P* < 0.05/11 = 0.004). Interestingly, we observed that two novel signals without cross-ancestry replication did demonstrate association with one or more lipid levels. For example, the AA associated *TMX2* intron variant rs518804 was validated based on its association with HDL and Triglycerides (HDL: 1.96 x 10^−06^ and Triglycerides: 0.001), while the LA associated *MARK2* intron variant rs10751002 was validated based on its association with LDL and Total Cholesterol (LDL: 3.31 x 10^−12^ and Total Cholesterol: 5.74 x 10^−09^) (Table 4, **Table S7 and Table S8**).

### Integrative Analyses Identify Putative Causal Genes For The Pufa Loci

Using colocalization with eQTL resources, we identified candidate genes underlying the genetic association signals for the PUFA traits. In HIS, we found colocalization with expression of the genes *MED19, TMEM258, PACS1, RAD9A, C11orf24, CTTN* on chromosome 11 and *PDXDC1* on chromosome 16 based on MESA multi-ancestry eQTL resources^26^ (Table 5 **and Table S9**). In further analysis using eQTL resources from GTEx whole blood, we confirmed colocalization with *TMEM258* and *MED19* identified using the MESA multi-ancestry eQTLs, and also identified colocalization with *FADS1, RPS4XP13, AP001462.2, PGA5, PGA5, TPCN2, MEN1* on chromosome 11 and *RP11-156C22.5* on chromosome 16. (Table 5 **and Table S10**).

Table 5 shows the results of integrative analysis including Colocalization analysis and PrediXcan in the HIS by using MESA data and GTEx data. For Colocalization analysis, eQTL resources include MESA multiethnic eQTL from purified monocytes and GTEx European ancestry whole blood tissue eQTL. GWAS signals with posterior colocalization probability of hypothesis 4 (PP.H4) > 0.80, or PP.H4 > 0.50 and the ratio of PP.H4 / PP.H3 > 5 were considered colocalized with eQTL. For PrediXcan, reference gene expression prediction models include MESA purified monocytes and GTEx European ancestry whole blood. Multiple testing correction of PrediXcan was applied for all genes (MESA: P < 0.05/4470 = 0.00001 and GTEx: P < 0.05/4350 = 0.00001).

We also performed complementary integrative analysis using PrediXcan, identifying significant associations for predicted expression of *TMEM258* with AA, ALA, DGLA, DPA, EPA, GLA and LA (after multiple testing correction for all genes examined: *P* < 0.05/4470 = 0.00001), based on integration with eQTL from both MESA and GTEx. PrediXcan also identified *TMEM109, ZBTB3, TTC9C, POLD4, INCENP* and *FERMT3* on chromosome 11 and *PDXDC1* on chromosome 16 as putative genes associated with PUFAs in HIS (Table 5, **Table S11 and Table S12**). For AFA, colocalization and PrediXcan analyses did not identify any genes of interest that met our pre-specified thresholds for statistical significance.

Incorporating the prior chromatin contacts identified (**Table S4**), we found that several of our GWAS regions had physical contact with one or more genes identified by integration with eQTL resources. For example, *RAD9A* was supported by colocalization with MESA eQTL and also showed chromatin contact with *POLD4* in nearly all cell types examined (Fig. 3B). In addition, *INCENP* was supported by PrediXcan using both MESA and GTEx resources and also showed chromatin contact with *TMEM258, FADS1* and *FADS2* in nearly all cell types examined (Fig. 3A). We further observed that *CLCF1*, *RAD9A, FADS2, TMEM258, INCENP, FADS1* identified from colocalization or PrediXcan were additionally supported by chromatin contacts analyses (**Table S4**, Fig. 3A and 3B).

To follow-up on the genes of interest identified by colocalization and PrediXcan analyses, we examined their co-expression with *FADS1* using GTEx whole blood gene expression with multiple testing correction for the number of genes under consideration (HIS: P < 0.05/39 = 0.0012). In both unadjusted and age/sex-a djusted regression models, multiple genes showed statistically significant co-expression with *FADS1*, for example, *TMEM258, MED19, POLD4, RAD9A* and *SSH3* (**Table S13**), suggesting these genes have shared patterns of expression.

## Discussion

To address the relative lack of prior studies examining genetics of PUFA levels in non-European ancestry populations, we carried out a meta-analysis of GWAS of n-3 and n-6 PUFAs in HIS and AFA across three cohorts: MESA, CHS and FHS. Examining genetic variants identified in prior CHARGE GWAS of the same traits in European Americans, we demonstrated evidence of association with n-3 and n-6 PUFAs for the signals in/near *FADS1/2* on chromosome 11, *PDXDC1* on chromosome 16, and *GCKR* on chromosome 2 in both HIS and AFA from our current CHARGE GWAS, as well as for *ELOVL2* on chromosome 6 in AFA only.

Through genome-wide analysis and subsequent statistical fine-mapping of our ancestry-specific results, we demonstrated evidence of multiple independent novel signals within the *FADS1/2* locus in both HIS and AFA, and in/near *ELOVL2* in AFA. Among these independent novel signals, we found one of the novel signals for LA identified in HIS demonstrated evidence of replication in AFA based on association with the same PUFA traits in MESA and CHARGE (HIS: rs11039018 intronic to *LRP4* [LDL receptor related protein 4]). This finding is supported by animal studies showing that deficiency of *Lrp4* in adipocytes increased glucose and insulin tolerance and reduced serum fatty acids.^30^ Additionally, multiple novel signals without cross-ancestry replication did show evidence of validation based on association with lipid levels in GLGC. For example, rs518804, a *TMX2* (thioredoxin related transmembrane protein 2) intron variant associated with AA and LA was validated based on its association with HDL and Triglycerides, while a *MARK2* (microtubule affinity regulating kinase *2)* intron variant rs10751002 associated with LA was validated based on its association with LDL and Total Cholesterol.

While we identified one signal in HIS with evidence of cross-ancestry replication, we also found a large number of signals in HIS that could not be replicated across race/ancestry groups (European, African American and Chinese), in part to differences in allele frequencies. For example, the chromosome 11 *POLD4* (DNA polymerase delta 4, accessory subunit) missense variant rs28364240 and *ANKRD13D* (ankyrin repeat domain 13D) intron variant rs142068305 identified in association with AA have minor allele frequencies of 0.204 and 0.196 in HIS, compared to frequencies close to zero in other race/ancestry groups.

Examining the distance between the putative causal variants in different credible sets identified in HIS, we observed that the signals on chromosome 11 span a long range (57.5Mb ~ 67.1 Mb). The extended physical distance covered by these independent PUFA-associated variants, combined with their subsequent validation in association with selected lipid traits, suggests there may be long-range chromatin interactions or other forms of physical interaction that may have yielded distinct genetic associations across this region.^31^ Interestingly, prior studies have reported the *FADS* signal on chromosome 11 as primarily just one genetic signal.^19,20^ However, our study provides evidence of two more independent signals (*BEST1* and *DAGLA*) within this *FADS* region. In order to understand the chromatin interactions of the *FADS* region on chromosome 11, we used ATAC-seq peaks and chromatin loops to perform the chromatin contact analyses. We identified multiple genes from colocalization or PrediXcan also supported by chromatin contacts, including *CLCF1*, *RAD9A, FADS2, TMEM258, INCENP* and *FADS1*, providing support for the role of our identified genetic signals in regulating these genes. In addition, we observed evidence of chromatin contacts among multiple distinct credible sets identified based on our fine-mapping of genetic signals on chromosome 11. For example, the region surrounding rs2668898 near *BEST1* also showed evidence of physical contact with the *TMEM258, FADS1 and FADS2* region in multiple cell types and *TMEM258* also showed evidence of physical contact with the *FADS1 and FADS2* region. This support for physical contact among some of the multiple independent signals within the *FADS* region opens the possibility of coordinated regulation among these distinct genetic signals. Besides the *FADS* region, *POLD4* also showed evidence of physical contact with the *ANKRD13D* region in multiple cell types. The cell types examined for chromatin interaction correspond to pancreas, liver, and other cell types that could play a role in synthesis and regulation of fatty acids. While the cell types used to examine chromatin interactions are distinct from those used for our integrative eQTL analyses, the chromatin interaction results do provide support for the plausible role of the genes identified by colocalization and PrediXcan.

Through integrative analyses including colocalization analysis and PrediXcan and overlapping our GWAS of PUFA levels with selected eQTL resources, we identified putative candidate genes that may shed light on the functional mechanisms of our identified genetic association signals. On chromosome 11 containing the *FADS* genes, we identified overlap with eQTL for multiple other genes including *MED19* (Mediator Complex Subunit 19), *TMEM258* (Transmembrane Protein 258), *PACS1* (Phosphofurin Acidic Cluster Sorting Protein 1), *RAD9A* (RAD9 Checkpoint Clamp Component A) and *CTTN* (Cortactin) suggesting additional complexity within this region beyond the *FADS* genes. For the signals on chromosome 16 identified based on analyses of DGLA in HIS and AFA, in/near *NTAN1* and *PDXDC1*, our integrative PrediXcan analyses identified *PDXDC1* (Pyridoxal Dependent Decarboxylase Domain Containing 1) (but not *NTAN1*) as a putative gene for DGLA. Additionally, having identified association with AA in HIS for the *POLD4* missense variant rs28364240, our subsequent identification of *POLD4* (DNA Polymerase Delta 4, Accessory Subunit) based on the PrediXcan analyses brings additional support for this gene. To follow-up on the genes of interest identified by colocalization and PrediXcan analyses, we examined their co-expression with *FADS1* using GTEx whole blood gene expression. Multiple genes on chromosome 11 identified in our integrative analyses combining the GWAS of PUFAs with whole blood expression from GTEx showed evidence of co-expression with *FADS1*, for example, *TMEM258, POLD4, TMEM109* and *ZBTB3*. This finding suggests some genomic regions at a considerable distance from *FADS1* may play a role in regulating its expression, and ultimately influence circulating PUFA levels.

While our genetic association study of PUFA levels in HIS and AFA provides novel insights, our work has several limitations. First, while we have combined data from multiple CHARGE cohorts, the overall sample size of our study is still relatively small for a GWAS. Second, as we began this GWAS effort some years ago, our work makes use of older imputation panels based on the 1000 Genomes. We expect future work could leverage newer resources including imputation based on the Trans-omics for Precision Medicine (TOPMed) reference panel or newer whole genome sequence data from TOPMed^32^. Third, the circulating PUFA levels examined in this study are derived from heterogeneous sources (plasma phospholipids in MESA and CHS vs. erythrocytes in FHS), which could have resulted in heterogeneity of genetic associations across the included studies and overall loss of power. Finally, while our integration of GWAS with eQTL proved useful in some cases, our efforts were driven in part by the available resources. We made use of multi-ancestry eQTL resources based on purified monocytes in MESA, as we knew these resources were well-matched with our GWAS cohorts in terms of LD structure, although purified monocytes were likely not the most relevant cell type for our study. We complemented those efforts with whole blood eQTL from GTEx through which we were able to capture colocalization of *FADS1* that was not observed in MESA due to the lack of significant cis-eQTL for *FADS1*. This limitation underscores the need for more diverse ancestry eQTL resources across a wider range of tissues and cell types.

In summary, working with the CHARGE Consortium, we conducted the first consortium-based GWAS of circulating PUFA levels in HIS and AFA cohorts. Our study demonstrated evidence of shared genetic influences on PUFA levels across race/ancestry groups, and demonstrated for the first time the large number of distinct genetic association signals within a neighborhood of the well documented *FADS* region on chromosome 11.^19,20^ Our findings provide new insight into the complex genetics of circulating PUFA levels that reflect, in part, their response to evolutionary pressures across the course of human history.^33,34^ Overall, our study demonstrates the value of investigating complex trait genetics in diverse ancestry populations and highlights the need for continued efforts for expanded genetic association efforts in cohorts with genetic ancestry that reflects that of the general population within the United States and worldwide.

## Methods

### Study participants

The participants in this study were recruited from three population-based cohorts: the Multi-Ethnic Study of Atherosclerosis (MESA)^35^, the Cardiovascular Health Study (CHS) and the Framingham Heart Study (FHS). This manuscript focuses on HIS participants from MESA (N = 1,243) and FHS (N = 211) and AFA participants from MESA (N = 1472), CHS (N = 603) and FHS (N = 203).

### Ethical Review

All cohort participants gave written informed consent, including consent to participate in genetic studies. All studies received approval from local ethical oversight committees.

### Fatty Acid Measurements

Circulating PUFA levels were quantified from plasma phospholipids in MESA and CHS, and from erythrocytes in FHS. Details on measurement of the PUFAs are provided in the **Supplementary Methods.**

### Genotyping And Imputation

Each of the participating cohorts had genome-wide genotype data based on a GWAS array, followed by imputation based on the 1000 Genomes Phase 1 v3 (for CHS) or Phase 3 (for MESA and FHS) Cosmopolitan reference panel.^36^ Details on genotyping, quality control and imputation are provided in the **Supplementary Methods.**

#### Data transformation and detection of outliers in measured PUFA Levels.

After examining the raw phenotype distributions for each of the phenotypes of interest, we applied variable transform for traits exhibiting deviation from normality. Log-transformation was applied for ALA, EPA and GLA. In addition, outliers for all of the PUFA levels were identified by the limits of median +/− 3.5 * MAD’, where MAD’ is computed with a scale factor constant of 1.4826 [default for the mad() function in R]. The value of MAD’ = 1.4826 * MAD0 where MAD0 is the raw value of median absolute deviation (MAD). For all the PUFAs, outliers were winsorized to the value of (median +/− 3.5 * MAD’).

### Meta-analysis Of Genome-wide Association Study

Genome-wide association analysis was carried out separately in each cohort and stratified by race/ancestry with covariate adjustment for age, sex, study site and principal components of ancestry. Cohort-specific GWAS results were filtered using EasyQC based on minor allele count (MAC) > 6 and imputation R-squared > 0.3. Cohort-specific results were combined using weighted sum of z-score metaanalysis in METAL^37^ and filtered using Effective Heterozygosity Filter (effHET) > 60. A threshold of *P* < 5 x 10^−8^ was applied to identify genome-wide significant loci.

### Identification Of Novel Versus Previously Reported Variants

Variants previously documented in the CHARGE GWAS meta-analysis of n-3 (n = 8,866)^19^ and n-6 (n = 8,631)^20^ PUFAs in European ancestry cohorts were considered known for the current meta-analysis. Additionally, those variants demonstrating linkage disequilibrium (LD) R-squared > 0.2 with one or more previously reported GWAS variants were considered known. The remaining variants were considered novel in the current study.

### Statistical Fine-mapping Using Susie

For each chromosome with more than one genome-wide significant variant (at *P* < 5 x 10^−8^), we carried out statistical fine-mapping to identify the putative causal variants and estimate the number of independent signals. We used Sum of Single Effect model (SuSiE)^24^ to identify the credible set of putative causal variants, providing as input all variants with *P* < 5 x 10^−8^ from the meta-analysis results. For fine-mapping of signals identified in our meta-analysis of HIS and AFA, we used imputed genotype dosage for the same set of variants in MESA HIS and AFA, respectively. To select the parameter L (prior number of independent signals) for fine-mapping in SuSiE, DAP-G (Deterministic Approximation of Posteriors)^38^ was conducted to provide a starting value for L based on the number of credible sets that the threshold of posterior inclusion probability was greater than 0.95.

### Follow-up Replication And Validation Analyses

Following statistical fine-mapping, cross-ancestry replication analyses were conducted for the most highly supported putative causal variant from each credible set using data on n-3 and n-6 PUFAs from other race/ancestry groups. The resources for replication analyses included the following:

1. **European Americans (EUR):** 2344 self-reported European American participants from MESA (using 1000 Genomes Phase 3 imputation, for comparison with the current study), as well as summary statistics from the previously published CHARGE GWAS meta-analysis of n-3 (n = 8,866)^19^ and n-6 (n = 8,631)^20^ PUFAs based on imputation from the HapMap Phase I and II,
2. **African Americans (AFA):** summary statistics from the present GWAS of PUFAs in AFA to examine cross-ancestry replication of variants identified in the present GWAS of HIS,
3. **Hispanic Americans (HIS):** summary statistics from the present GWAS of PUFAs in HIS to examine cross-ancestry replication of variants identified in the present GWAS of AFA, and
4. **Chinese Americans (CHN):** 649 self-reported Chinese American participants from MESA (using 1000 Genomes Phase 3 imputation, for comparison with the current study).

Given the limited number of cohorts available for ethnic-specific and cross-ethnic replication of PUFA traits, additional validation analyses were conducted for the same set of variants using multi-ancestry genetic association with lipid traits (HDL, LDL, total cholesterol and triglycerides) from the Global Lipids Genetics Consortium (GLGC).^25^ Multiple testing correction was applied to account for the number of variants examined in cross-ethnic replication (HIS: *P* < 0.05/19 = 0.0026 and AFA: *P* < 0.05/11 = 0.004).

### Bayesian Colocalization Analysis

Bayesian colocalization analysis has proven an effective approach for identification of downstream genes underlying GWAS loci.^35^ We used the R/coloc package to conduct Bayesian colocalization analysis^39^ to identify the putative gene(s) corresponding to each credible set of variants using MESA multi-ancestry eQTL data from purified monocytes^26^ and GTEx multi-ancestry whole blood tissue eQTL data.^40^ Bayesian colocalization analysis tested the following hypotheses: H0. neither GWAS of PUFAs nor eQTL has a genetic association in the region (within 1 Mb of the transcription start site); H1. only GWAS of PUFAs has a genetic association in the region; H2. only eQTL has a genetic association in the region; H3. both GWAS of PUFAs and eQTL are associated, but with different causal variants; H4. both GWAS of PUFAs and eQTL are associated and share a single causal variant. Colocalization for variants in credible sets was defined by (1) a posterior colocalization probability of hypothesis 4 (PP.H4) > 0.80, or (2) a PP.H4 > 0.50 *and* the ratio of PP.H4 / PP.H3 > 5.

#### PrediXcan model.

PrediXcan, a gene-based association method focused on identifying trait-associated genes by quantifying the effect of gene expression on the phenotype on interest.^41^ In this study, we applied summary-statistics based PrediXcan (S-PrediXcan)^42^ using reference gene expression prediction models from MESA purified monocytes^26^ and GTEx multi-ancestry whole blood.^43^ S-PrediXcan associations were considered genomewide significant if they passed the multiple testing correction for all genes (MESA: *P* < 0.05/4470 = 0.00001 and GTEx: *P* < 0.05/4350 = 0.00001).

### Chromatin Contact Analysis

To identify variants located in open chromatin regions in contact gene promoters, we used GenomicRanges (v. 1.46.1 ; R version 4.1.1) to intersect the genomic coordinates (hg19) of the variants contained in the credible sets with the open chromatin peaks (called using the ENCODE pipeline) in significantly enriched contact with gene promoter determined by Promoter Capture C (Chicago Score > 5). We queried chromatin accessibility and promoter contacts in human mesenchymal stem cells (hMSC) and Adipocytes differentiated in vitro from these (hMSC_Adipocytes), embryonic stem cell derived hypothalamic neurons (hESC Hypothalamic Neurons), induced pluripotent-dervived Heptocytes (IPS-Hepatocytes), Enteroids, and the hepatic carcinoma HepG2 cell line.^44-49^ Details on Promoter Capture C and ATAC-seq library generation and analyses have been previously described.^44^

#### Gene Co-expression Analysis.

We used the GTEx whole blood gene expression version 8 TPM dataset to examine co-expression with *FADS1* for genes identified by integrative analyses, including colocalization and PrediXcan. Two models for gene co-expression analysis were used for the trait of interest,

1. an unadjusted model *FADS1* ~ gene expression; and
2. a covariate adjusted model *FADS1* ~ age + gender + gene expression.

Gene co-expression associations were considered statistically significant if they passed the multiple testing correction for all genes examined from colocalization and PrediXcan (*P* < 0.05/39 = 0.0012).

## Figures and Tables

**Table 1:** CHARGE cohorts descriptives

	MESA/Hispanic Americans	FHS/Hispanic Americans	MESA/African Americans	CHS/African Americans	FHS/African Americans
**Participant characteristics**					
No. subjects	1243	211	1472	603	203
Women	629 (50.6)	129 (61.1)	788 (53.5)	390 (64.7)	130 (64.0)
Age, years	61 [53, 69]	53 [44, 60]	63 [53, 70]	74 [71, 79]	58 [50, 67]
**n-3 Polyunsaturated Fatty Acids**					
ALA (% of total fatty acids)	0.16 [0.12, 0.20]	0.21 [0.16, 0.27]	0.15 [0.12, 0.19]	0.13 [0.11, 0.17]	0.18 [0.15, 0.23]
EPA	0.53 [0.37, 0.74]	0.57 [0.47, 0.78]	0.68 [0.51, 0.98]	0.53 [0.39, 0.67]	0.68 [0.48, 1.01]
DPA	0.86 [0.73, 1.00]	2.49 [2.13, 2.79]	0.93 [0.80, 1.07]	0.85 [0.75, 0.97]	2.54 [2.25, 2.89]
DHA	2.96 [2.29, 3.77]	4.21 [3.45, 5.13]	4.05 [3.25, 4.95]	3.46 [2.87, 4.17]	5.23 [4.21, 6.47]
**n-6 Polyunsaturated Fatty Acids**					
LA	20.92 [18.87, 23.07]	14.32 [12.24, 16.76]	18.88 [17.12, 20.84]	17.84 [16.46, 19.40]	12.53 [10.88, 15.16]
GLA	0.11 [0.08, 0.14]	0.15 [0.10, 0.18]	0.10 [0.08, 0.13]	0.07 [0.05, 0.09]	0.10 [0.07, 0.15]
DGLA	3.57 [3.04, 4.13]	1.95 [1.63, 2.35]	2.89 [2.47, 3.33]	2.76 [2.39, 3.24]	1.51 [1.32, 1.78]
AA	11.01 [9.37, 12.84]	16.56 [15.17, 17.74]	13.21 [11.65, 14.82]	12.64 [11.57, 13.86]	17.17 [15.95, 18.48]

**Table 2:** Genome-wide significant signals (Credible sets) for PUFAs in CHARGE Hispanic Americans.

	Variants (Chr:Pos:EFF:OTH)	EAF	Zscore	Pvalue	Cluster	#Of SNP	Novel/ Known	Nearest Gene
**AA**	rs102274 (11:61557826:C:T)	0.506	−24.26	5.1E-130	1	7	Known	*TMEM258*
	rs142068305 (11:67065755:T:G)	0.196	−7.06	1.63E-12	2	1	Novel	*ANKRD13D*
	rs28364240 (11:67120530:G:C)	0.204	−7.04	1.88E-12	3	1	Novel	*POLD4*
	rs2668898 (11:61725498:G/A)	0.402	−5.83	5.32E-09	4	1	Known	*BEST1*
	rs180792704 (11:67325239:C:G)	0.199	−7.56	3.81E-14	5	1	Novel	*NA*
	rs198434 (11:61483417:A:G)	0.710	−8.97	2.80E-19	6	1	Known	*DAGLA*
	rs518804 (11:57494487:C:A)	0.420	−7.73	1.01E-14	7	1	Novel	*TMX2*
	rs3177514 (11:66130358:G:T)	0.699	−5.60	2.06E-08	8	1	Novel	*SLC29A2*
**ALA**	rs174562 (11:61585144:G:A)	0.503	7.84	4.30E-15	1	23	Known	*FADS1*
**DGLA**	rs174538 (11:61560081:A:G)	0.488	14.70	6.03E-49	1	1	Known	*TMEM258*
	rs174585 (11:61611694:A:G)	0.274	9.82	8.72E-23	2	1	Known	*FADS2*
	rs198434 (11:61483417:A:G)	0.710	6.27	3.57E-10	3	1	Known	*DAGLA*
	rs198461 (11:61524366:C:A)	0.363	−5.95	2.54E-09	4	1	Known	*MYRF*
	rs57112407 (15:78088914:T:C)	0.255	−5.86	4.46E-09	NA	NA	Novel	*LINGO1*
	rs4985155 (16:15129459:G:A)	0.524	−7.72	1.16E-14	1	25	Known	*PDXDC1*
**DPA**	rs1535 (11:61597972:G:A)	0.520	−11.31	1.07E-29	1	18	Known	*FADS2*
	rs198434 (11:61483417:A:G)	0.710	−6.26	3.67E-10	2	1	Known	*DAGLA*
**EPA**	rs102274 (11: 61557826:C:T)	0.506	−11.56	6.18E-31	1	17	Known	*TMEM258*
**GLA**	rs174576 (11: 61603510:A:C)	0.546	−7.73	1.07E-14	1	19	Known	*FADS2*
**LA**	rs174564 (11:61588305:G:A)	0.520	15.11	1.23E-51	1	10	Known	*FADS2*
	rs10751002 (11:63617634:G:T)	0.664	6.06	1.36E-09	2	1	Novel	*MARK2*
	rs2668898 (11:61725498:G:A)	0.402	5.54	2.99E-08	3	1	Known	*BEST1*
	rs28364240 (11:67120530:G:C)	0.204	5.90	3.44E-09	4	1	Novel	*POLD4*
	rs11039018 (11:46909524:A:C)	0.67	−6.10	1.01E-09	5	1	Novel	*LRP4*
	rs518804 (11:57494487:C:A)	0.420	6.03	1.62E-09	6	1	Known	*TMX2*

**Table 3:** Genome-wide significant signals (Credible sets) for PUFAs in CHARGE African Americans.

	Variants (Chr:Pos:EFF:OTH)	EAF	Zscore	Pvalue	Cluster	#Of SNP	Novel/ Known	Nearest Gene
**AA**	rs174585 (11:61611694:A:G)	0.060	−9.32	1.08E-20	1	1	Known	*FADS2*
	rs174607 (11:61627321:C:G)	0.078	−6.49	8.47E-11	2	1	Known	*FADS2*
	rs174564 (11:61588305:G:A)	0.133	−14.85	6.43E-50	3	1	Known	*FADS2*
	rs174559 (11:61581656:A:G)	0.078	−13.68	1.27E-42	4	1	Known	*FADS1*
	rs17161592 (7:9388418:C:G)	0.085	−6.31	2.75E-10	1	2	Novel	*NA*
**DGLA**	rs174560 (11:61581764:C:T)	0.216	9.12	7.51E-20	1	1	Known	*FADS1*
	rs1136001 (16:15131974:T:G)	0.220	−6.11	9.69E-10	2	17	Known	*PDXDC1*
**DPA**	rs717894 (6:22119292:A:G)	0.250	−5.48	4.11E-08	1	1	Novel	*CASC15*
	rs9295741 (6:10997166:T:C)	0.223	5.54	2.89E-08	2	2	Known	*ELOVL2*
**DHA**	rs114622288 (10:14663844:A:G)	0.050	−5.71	1.16e-08	NA	NA	Novel	*FAM107B*
**LA**	rs28456 (11:61597972:G:A)	0.163	7.88	3.14E-15	1	2	Known	*FADS2*

**Table 4:** Novel PUFA-associated signals (credible sets) from analysis of HIS with external replication or validation evidence.

Traits	Variants (chr:pos:effect:other)	Replication	Validation	Direction	Nearest Gene
AA	rs518804 (11:57494487:C:A)	NS	HDL: P = 1.96E-06 logTG: P = 0.001	HDL: (−) LDL: (−) logTG: (+)	*TMX2*
LA	rs10751002 (11:63617634:G:T)	NS	LDL: P = 3.31E-12 TC: P = 5.74E-09	LDL: (+) TC: (+)	*MARK2*
	rs11039018 (11:46909524:A:C)	AFA: P = 1.90E-13	HDL: P = 2.85E-74 logTG: P = 4.5E-43	AFA: (+) HDL: (+) logTG: (−)	*LRP4*

**Table 5:** Integrative analysis (Colocalization and PrediXcan) in the Hispanic Americans using multi-ancestry resources from MESA and GTEx.

	Colocalization Analysis		PrediXcan	
	MESA multi- ethnics eQTLs	GTEx eQTLs	MESA	GTEx
**AA**	Chromosome 11			
	*MED19, TMEM258, PACS1, RAD9A*	*RPS4XP13, AP001462.6*	*TMEM258, TMEM109, ZBTB3, TTC9C, FERMT3, MED19, POLD4, CLCF1, INCENP, MADD, SSH3, C11orf24, PRPF19, TBC1D10C, BANF1, CCDC86, NXF1, MS4A6E, CCS, COX8A, CCDC88B, ACP2, MAP4K2*	*TMEM258, TMEM223, NXF1, INCENP, MUS81, C11orf84, MED19, MEN1, BBS1, NEAT1, DPP3, SSH3, ACP2, ASRGL1, RNASEH2C*
**ALA**	Chromosome 11			
	*TMEM258, MED19*	*MED19, PGA5, TMEM258*	*TMEM258, TMEM109*	*TMEM258*
**DGLA**	Chromosome 11			
	*TMEM258*		*TMEM258, ZBTB3*	*TMEM258, FADS1, FADS2*
	Chromosome 16			
	*PDXDC1*	*RP11-426C22.5*	*PDXDC1*	*NPIPA2*
**DPA**	Chromosome 11			
	*TMEM258, C11orf24, RAD9A*	*PGA5*	*TMEM258, TMEM109*	*TMEM258, SSH3, TMEM223*
**EPA**	Chromosome 11			
	*TMEM258*	*TPCN2*	*TMEM258, FERMT3, TMEM109*	*TMEM258, SSH3, TMEM223*
**GLA**	Chromosome 11			
	*TMEM258*	*MEN1*	*TMEM258*	*TMEM258*
	Chromosome 11			
**LA**	*MED19, CTTN, C11orf24, RAD9A*	*MED19, TPCN2, FADS1, RPS4XP13, AP001462.6*	*TMEM258, TMEM109, FERMT3, ZBTB3, COX8A, MADD, POLD4, TBC1D10C, INCENP, TTC9C, MED19, CLCF1, SSH3, ACP2*	*TMEM258, INCENP, SSH3, C11orf84, TMEM223, GIF, NXF1, MED19, MUS81, ACP2*
